# Validating *HMMR* Expression and Its Prognostic Significance in Lung Adenocarcinoma Based on Data Mining and Bioinformatics Methods

**DOI:** 10.3389/fonc.2021.720302

**Published:** 2021-08-30

**Authors:** Xia Li, Haiwei Zuo, Li Zhang, Qiuwen Sun, Yong Xin, Longzhen Zhang

**Affiliations:** ^1^First Clinical College, Xuzhou Medical University, Xuzhou, China; ^2^Department of Radiation Oncology, Affiliated Hospital of Xuzhou Medical University, Xuzhou, China; ^3^School of Medical Information & Engineering, Xuzhou Medical University, Xuzhou, China; ^4^School of Information and Control Engineering, China University of Mining and Technology, Xuzhou, China; ^5^School of Medical Imaging, Xuzhou Medical University, Xuzhou, China; ^6^Cancer Institute, Xuzhou Medical University, Xuzhou, China

**Keywords:** lung adenocarcinoma, *HMMR*, TCGA, bioinformatics, analysis, GSEA, PPI, prognosis

## Abstract

Hyaluronic acid-mediated motility receptor (*HMMR*), a tumor-related gene, plays a vital role in the occurrence and progression of various cancers. This research is aimed to reveal the effect of *HMMR* in lung adenocarcinoma (LUAD). We first obtained the gene expression profiles and clinical data of patients with LUAD from The Cancer Genome Atlas (TCGA) database. Then, based on the TCGA cohort, the *HMMR* expression difference between LUAD tissues and nontumor tissues was detected and verified with public tissue microarrays (TMAs), clinical LUAD specimen cohort, and Gene Expression Omnibus (GEO) cohort. Logistic regression analysis and chi-square test were adopted to study the correlation between *HMMR* expression and clinicopathological parameters. The effect of *HMMR* expression on survival was evaluated by Kaplan–Meier survival analysis and using the Cox regression model. Furthermore, Gene Set Enrichment Analysis (GSEA) was utilized to screen out signaling pathways related to LUAD and the co-expression analysis was employed to build the protein–protein interaction (PPI) network. The *HMMR* expression level in LUAD tissues was dramatically higher than that in nontumor tissues. Logistic regression analysis and chi-square test demonstrated that the high *HMMR* expression in LUAD has relation with gender, pathological stage, T classification, lymph node metastasis, and distant metastasis. The Kaplan–Meier curve suggested a poor prognosis for LUAD patients with high *HMMR* expression. Multivariate analysis implied that the high *HMMR* expression was a vital independent predictor of poor overall survival (OS). GSEA indicated that a total of 15 signaling pathways were enriched in samples with the high *HMMR* expression phenotype. The PPI network gave 10 genes co-expressed with *HMMR*. *HMMR* may be an oncogene in LUAD and is expected to become a potential prognostic indicator and therapeutic target for LUAD.

## Background

According to the GLOBOCAN 2018 estimation, there were more than 2,000,000 new cases of lung cancer all over the world, accounting for 11.6% of new cancer cases, which makes lung cancer the most common malignant tumor on earth (1). Approximately 1,700,000 patients died of lung cancer, accounting for 18.4% of total cancer-related deaths (2). Thus, lung cancer is considered to be the main cause of cancer-related deaths in global world. Non-small cell lung cancer (NSCLC) patients account for nearly 85% of all lung cancer cases, and almost 50% of them suffer from LUAD, which is the most common lung malignancy (3). In the past two decades, significant advancements have been achieved in the treatment of NSCLC, including tyrosine kinase inhibitors and immunotherapy (4). However, persistent reactions are rare and the prognosis is still very poor, with a 19% overall 5-year survival rate in the United States and a worldwide ratio of lung cancer mortality-to-incidence of 0.87 (5). Consequently, it is essential to determine a biomarker that can forecast the prognosis of LUAD with high sensitivity and strong specificity and act as a target for LUAD treatment.

Hyaluronan, an extracellular matrix component, can not only absorb water in tissues but also regulate the proliferation of stem cell populations (6). It was reported that hyaluronan receptors were highly expressed in stem cells isolated from normal tissues (7). *HMMR*, a protein related to centrosomes and microtubules, is one of several well-defined receptors for hyaluronan (8). On the one hand, the central region of human *HMMR* is a coiled-coil stem, which can serve as a potential dimerization domain and binding region for other proteins (9). Specifically, the interaction between *HMMR* and *CHICA/FAM83D* is based on amino acids 365–546 (10), and amino acids 574–602 can act as calcium-dependent calmodulin-binding domains (11). On the other hand, the two microtubule-binding subdomains of human *HMMR* are located at amino acids 40–59 and 76–90, respectively (12). Besides, the microtubule-binding subdomain at amino acids 76–90 is encoded by exon 4 (12). On the basis of the domains, *HMMR* can directly bind to microtubules (12). Moreover, the conserved C-terminal bZip motif in *HMMR* overlaps with the designated B-X7-B motif, and the two motifs bind to hyaluronic acid and heparin in an ionic manner (13). Because of the structural characteristics and chemical properties, *HMMR* is capable of binding to microtubules *via* its N-terminal and localizing to the centrosome by virtue of its C-terminal bZip motif. Also, serving as a binding partner of different spindle assembly factors, *HMMR* can regulate the assembly, stability, and position of spindle microtubules during mitosis and meiosis (14).

In recent years, a growing number of scholars at home and abroad have begun to devote themselves to studying the mechanism of *HMMR* in the occurrence and development of LUAD. Several representative studies are as follows. With the aim of understanding potential mechanisms of LUAD recurrence, Stevens et al. (15) studied the network of extracellular matrix (ECM) molecules by analyzing Affymetrix data and RNA-seq data. It turned out that the overexpression of *HMMR* in primary LUAD had associations with a poor prognosis (15). Further, reducing *HMMR* in LUAD cells could decrease their abilities to induce lung cancers and distant metastases (15). Afterward, through utilizing the step-wise multivariate Cox analysis, He et al. (16) developed a novel eight-gene prognostic signature (*CDCP1*, *HMMR*, *TPX2*, *CIRBP*, *HLF*, *KBTBD7*, *SEC24B-AS1*, and *SH2B1*) for early-stage NSCLC. This signature might be helpful to personalized treatment decisions. Furthermore, applying the data-mining method, Liu et al. (17) designed a risk score staging system to predict the prognosis of LUAD patients. They identified a four-gene signature (*AGRN*, *AKR1A1*, *DDIT4*, and *HMMR*) to divide LUAD patients into the high-risk group and the low-risk group. Analogously, utilizing the mRNA-mining method to analyze mRNA expression profiling in the large LUAD cohorts, Zhang et al. (18) confirmed a total of nine genes (*HMMR*, *B4GALT1*, *SLC16A3*, *ANGPTL4*, *EXT1*, *GPC1*, *RBCK1*, *SOD1*, and *AGRN*) that were significantly associated with metastasis and OS in LUAD patients.

Although some studies have explored potential associations between *HMMR* and LUAD, known evidence for *HMMR* as a biomarker is still insufficient and the specific mechanism of *HMMR* is still unclear in LUAD. Based on the TCGA database and GEO database, this research has investigated the associations between the *HMMR* expression level and the clinicopathological characteristics of LUAD, as well as the prognostic significance of *HMMR*, in order to provide more evidence for the potential role of *HMMR* in LUAD. Then, GSEA was implemented to deepen the understanding of the signal pathways involved in *HMMR* regulatory networks related to LUAD. Meanwhile, the PPI network was constructed to predict genes co-expressed with *HMMR*.

## Materials and Methods

### RNA-Sequencing and Clinical Data From TCGA

On or before October 7, 2020, the raw gene expression data for 497 LUAD tissues and 54 adjacent nontumor tissues were obtained from the TCGA database. The workflow type of each LUAD case is HTSeq-FPKM. In the same way, we obtained the raw expression data of common cancers. The corresponding clinical data of LUAD patients were also achieved. These clinical data contain information of age, gender, pathological stage, T stage, N stage, M stage, and vital status (**Table 1** **)**. This research is in full compliance with the guidelines of the National Institute of Health (NIH) TCGA human subject protection and data access policies.

**Table 1:** Characteristics of patients with LUAD.

Characteristics	Variable	Patients (486)	Percentages (%)
**Age**	<65 years	209	43.00
	≥65 years	258	53.09
	Unknown	19	3.91
**Gender**	Male	222	45.68
	Female	264	54.32
**Pathological stage**	I	262	53.91
	II	112	23.05
	III	79	16.25
	IV	25	5.14
	Unknown	8	1.65
**T classification**	T1	163	33.54
	T2	260	53.50
	T3	41	8.44
	T4	19	3.91
	TX	3	0.61
**N classification**	N0	312	64.20
	N1	90	18.52
	N2	70	14.40
	N3	2	0.41
	NX	12	2.47
**M classification**	M0	333	68.52
	M1	24	4.94
	MX	129	26.54
**Vital status**	Alive	324	66.67
	Death	162	33.33

### Fresh Frozen Tissue Specimen Cohort

From March 19, 2021, to April 4, 2021, we collected 32 pairs of fresh frozen LUAD tissues and adjacent nontumor tissues at the Affiliated Hospital of Xuzhou Medical University. These samples were preserved at -80°C for quantitative real-time PCR (qRT-PCR). The project was granted approval by the Ethics Committee of the Affiliated Hospital of Xuzhou Medical University.

### Online Bioinformatics Analysis

Two online public available database including TIMER2.0 (http://timer.cistrome.org/) and UALCAN (http://ualcan.path.uab.edu/) were utilized to observe the mRNA and protein levels of *HMMR* in human pan-cancer. The PPI network for *HMMR* was constructed and visualized based on the STRING database (http://string-db.org/). The correlation of mRNA level between *HMMR* and its co-expressed genes in the PPI network was analyzed by the GEPIA online database (http://gepia.cancer-pku.cn/).

### *HMMR* Expression and Survival Analysis

First, Perl was applied to process raw gene expression data from the TCGA database. Through employing the limma package, *HMMR* expression data were extracted from processed data. To visualize *HMMR* expression data, the limma package and beeswarm package were utilized to draw scatter difference chart and paired difference chart. The survival data of patients with LUAD were extracted from downloaded clinical information, and samples without survival time or survival status information were filtered out. We matched the complete survival information of each sample with its *HMMR* expression data and then received 458 LUAD samples who meet requirements. According to the *HMMR* expression median, 458 LUAD patients were divided into two groups (high *HMMR* expression group and low *HMMR* expression group). Based on the two groups, we drew the Kaplan–Meier survival curve by utilizing the survival package.

### RNA Extraction and qRT-PCR

According to instructions, we utilized TRIzol reagent (Invitrogen) to extract total RNA from fresh frozen LUAD tissues and adjacent nontumor tissues. Then, TransScript One-Step Guide DNA Removal and Complementary DNA Synthesis SuperMix were used for the reverse transcription reaction. The primer sequences for PCR amplification were as follows: *HMMR*, forward: 5′-AACAAGCTGAAAGGCTGGTCA-3′, reverse: 5′-GGGTATGAGCAGCACTACTTTT-3′.

### Verification of *HMMR* From GEO and Human Protein Atlas

Adopting “lung” and “adenocarcinoma” as search terms, and “Homo sapiens” as the qualifier, we searched microarrays that met experimental requirements in the GEO database. After excluding datasets with a sample size of less than 40, there were still seven eligible datasets (GSE101929, GSE11969, GSE18842, GSE21933, GSE27262, GSE32863, and GSE75037). As shown in **Table 2**, the selected datasets included 359 LUAD tissue samples and 271 nontumor tissue samples. Based on Review Manager 5.3 software, the meta-analysis was applied to evaluate the differences of *HMMR* expression between LUAD samples and nontumor samples. We calculated the combined value on the basis of standard mean difference (SMD) with a 95% confidence interval (CI). Meanwhile, the heterogeneity between seven selected datasets was assessed by chi-square (*χ* ^2^) and *I* ^2^ statistical tests. If *p* > 0.05 or *I* ^2^ < 50%, a fixed-effect model was chosen to calculate the combined effect; otherwise, a random effect model was selected (*p* < 0.05 or *I* ^2^ > 50%). What is more, taking “*HMMR*,” “lung,” and “adenocarcinoma” as search terms, we also obtained immunohistochemical results of patients with LUAD from the human protein atlas database (http://www.proteinatlas.org).

**Table 2:** Information of selected GEO series datasets.

GEO datasets	Year	Country	Platform	Sample	N
**GSE101929**	2019	USA	GPL570	LUAD	32
				Non-LUAD	34
**GSE11969**	2013	Japan	GPL7015	LUAD	94
				Non-LUAD	5
**GSE18842**	2019	Spain	GPL570	LUAD	46
				Non-LUAD	45
**GSE21933**	2014	China	GPL6254	LUAD	21
				Non-LUAD	21
**GSE27262**	2019	China	GPL570	LUAD	25
				Non-LUAD	25
**GSE32863**	2019	USA	GPL6884	LUAD	58
				Non-LUAD	58
**GSE75037**	2019	USA	GPL6884	LUAD	83
				Non-LUAD	83

### Univariate and Multivariate Cox Regression Analyses

Based on the TCGA dataset and Cox regression model, we conducted univariate and multivariate analyses and calculated the hazard ratio (HR) and 95% CI. Simultaneously, we performed a quantitative assessment of the predictive value of clinicopathological parameters and *HMMR* expression on survival. *Via* adjusting for confounding factors, the independent prognostic effect of *HMMR* on survival was estimated. Concretely, Perl was utilized to process raw clinical data of LUAD patients and delete samples with incomplete clinical information. Then, the processed clinical data were matched with the *HMMR* expression data and LUAD patients were divided into the high *HMMR* expression group or the low *HMMR* expression group according to the *HMMR* expression value. Finally, the data of 316 LUAD patients were applied to perform univariate and multivariate Cox regression analyses.

### Gene Set Enrichment Analysis

GSEA is used to confirm whether a given set of genes displays statistically obvious and consistent differences between two biological states (19). The GSEA software was adopted to seek signaling pathways associated with *HMMR* between datasets with low or high *HMMR* expression. In GSEA software, the annotated gene set (c2.cp.kegg.v6.2.symbols.gmt) was regarded as the reference gene set. In each analysis, we set 1,000 genes to identify distinct pathways and gene-set permutations were implemented 1,000 times. The normalized enrichment score (NES), nominal *p*-value, and false discovery rate (FDR) *q*-value were given to denote the importance of associations between gene sets and pathways.

### Statistical Analysis

A variety of statistical methods were adopted to achieve a comprehensive analysis. The Mann–Whitney U test was utilized to detect *HMMR* expression differences between LUAD tissue samples and nontumor tissue samples. The Kruskal–Wallis test was applied to inspect *HMMR* expression differences among multiple groups. The correlation between *HMMR* expression and each clinicopathological parameter was evaluated through the *χ* ^2^ test. The log-rank test was performed to compare the survival rate difference between the high *HMMR* expression group and the low *HMMR* expression group. The Cox regression model was employed to carry out univariate and multivariate survival analyses. In these statistical analysis methods, we adopted *p* < 0.05 to determine the significance level.

## Results

### *HMMR* Is Upregulated in Pan-Cancer and LUAD According to Public Databases

**Figure 1:**
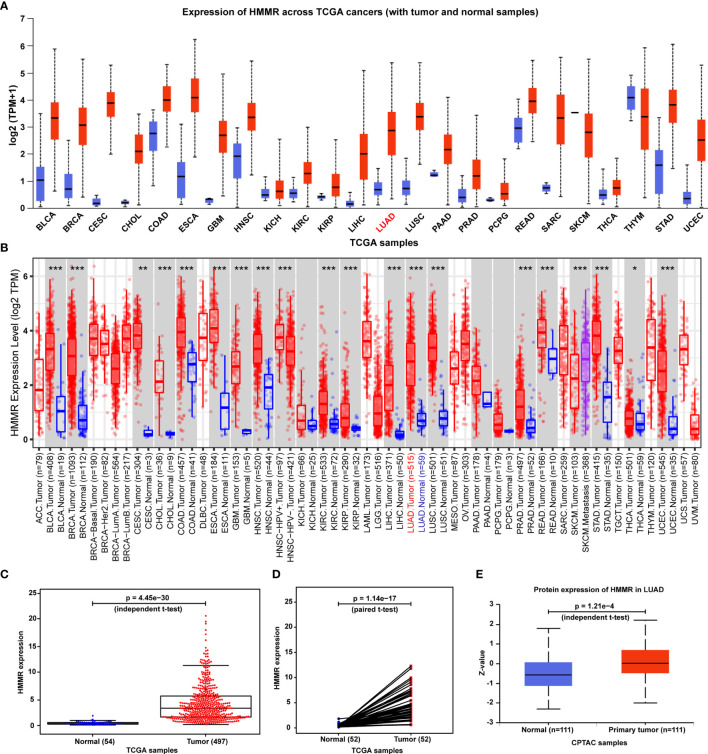
*HMMR* expression in normal human tissues, human tumors, and LUAD. **(A)** *HMMR* mRNA expression between multiple human cancers and corresponding normal tissues in the UALCAN database. **(B)** *HMMR* expression level among a variety of human cancers in the TIMER database (*p < 0.05, **p < 0.01, ***p < 0.001). **(C)** *HMMR* mRNA expression between tumors and normal tissues of LUAD patients from the TCGA database. **(D)** *HMMR* mRNA expression between pairs of tumors and normal tissues of LUAD patients from the TCGA database. **(E)** *HMMR* protein expression between primary tumors and normal tissues of LUAD patients from the CPTAC database.

To determine whether *HMMR* is involved in human cancers, we first analyzed the expression of *HMMR* in different types of cancers *via* utilizing the UALCAN database. We observed that *HMMR* mRNA expression was significantly higher in most human cancers compared with the corresponding normal tissues (**Figure 1A**). Consistent with this result, *HMMR* was also found to be notably upregulated in numerous human solid tumors according to the TIMER 2.0 database (**Figure 1B**). Then, further analysis of *HMMR* mRNA expression was carried out for 551 tissues (497 LUAD tissues and 54 adjacent nontumor tissues) from the TCGA database. The LUAD tissues displayed a significantly higher *HMMR* mRNA expression level than the normal tissues (**Figure 1C**). What is more, the result was also validated by data from 54 tumor tissues and paired adjacent nontumor tissues of LUAD patients in the TCGA database (**Figure 1D**), and a CPTAC cohort containing 111 cancer patients and 111 adjacent nontumor tissues (**Figure 1E**).

Moreover, as seen in **Figure 1A**, *HMMR* is found to be overexpressed in various cancers. To further analyze the specificity of *HMMR* for LUAD diagnosis, we calculated the difference multiple of the *HMMR* expression between normal samples and tumor samples for common cancers. After filtering out these cancers whose samples cannot be obtained and the number of normal samples less than 10, we finally achieved the difference multiple of *HMMR* in 12 different cancers. As listed in **Table S1**, we observed that the difference multiple of the *HMMR* expression was greater than 3 in only three cancers (LUAD: log2FC=3.22, LUSC: log2FC=3.32, LIHC: log2FC=3.99). From the perspective of *HMMR* expression, *HMMR* has a certain specificity for LUAD diagnosis. The specificity of *HMMR* for LUAD diagnosis at the level of regulatory mechanisms will be explored in our future work.

### Validation of *HMMR* Upregulation in LUAD by Public TMAs, qRT-PCR, and SMD

**Figure 2:**
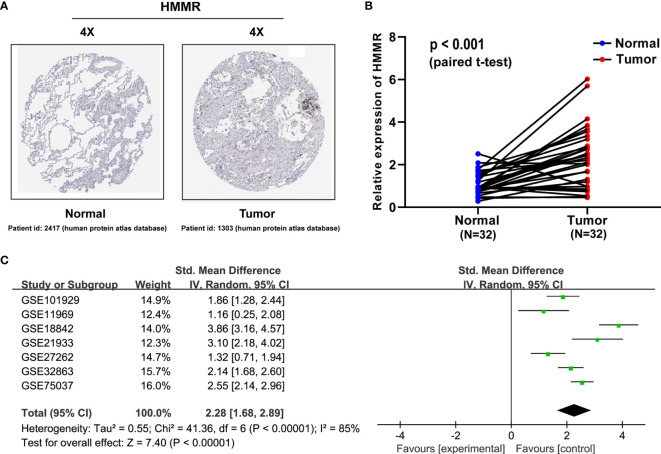
*HMMR* is upregulated in LUAD patient specimens. **(A)** *HMMR* expression in normal lung tissues and LUAD specimens. Images were taken from the human protein atlas (http://www.proteinatlas.org) online database. **(B)** Expression of *HMMR* in 32 LUAD tissues and corresponding nontumor tissues were detected by qRT-PCR (*p* < 0.001). **(C)** Forest plot of *HMMR* expression data from GEO microarrays. Gene Expression Omnibus (GEO), standard mean difference (SMD), confidence interval (CI).

To characterize the *HMMR* expression status, we analyzed the *HMMR* protein expression in clinical specimens from the human protein atlas database (http://www.proteinatlas.org). We found that *HMMR* had a positive expression in LUAD tissues and a negative expression in normal lung tissues (**Figure 2A**). To further verify the difference of the *HMMR* expression in the TCGA database, the *HMMR* mRNA expression was validated in a clinical *HMMR* cohort containing 32 pairs of fresh frozen tissue specimens, revealing that *HMMR* was upregulated in LUAD tissues compared with adjacent nontumor tissues (**Figure 2B**) (*p* < 0.001). Moreover, a comprehensive meta-analysis of *HMMR* expression data for LUAD patients in the GEO dataset (**Table 2**) was performed. As depicted in **Figure 2C**, the *I*-square value was 85% (*p* < 0.001) and the combined SMD of *HMMR* was 2.28 in view of the random-effect model (95% CI: 1.68–2.89). These clearly indicated that *HMMR* was highly expressed in LUAD.

### *HMMR* Is Associated With Malignant Progression in Patients With LUAD

**Figure 3:**
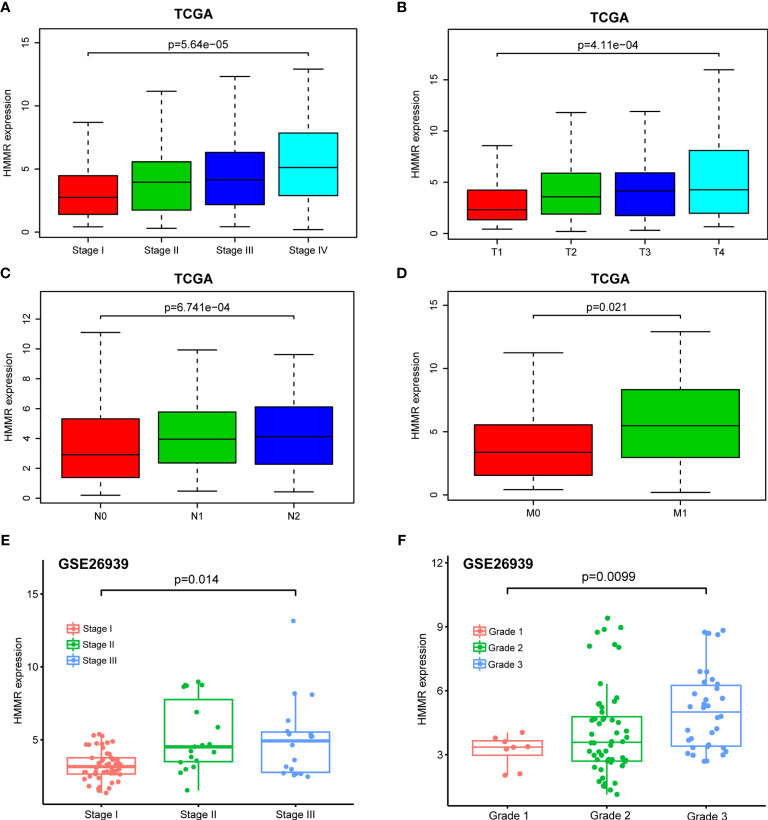
Box plot to evaluate *HMMR* mRNA expression in LUAD patients based on clinical characteristics. **(A)** Pathological stage. **(B)** T classification. **(C)** N classification. **(D)** M classification. **(E)** Pathological stage in GSE26939. **(F)** Histological grade in GSE26939.

With the in-depth study of *HMMR* mRNA expression data in the TCGA database, we were surprised to find that the expression of *HMMR* was different in groups classified according to pathological stage (*p* < 0.001, **Figure 3A** and *p* < 0.05, **Figure 3E** **)**, T classification (*p* < 0.001, **Figure 3B** **)**, N classification (*p* < 0.001, **Figure 3C** **)**, M classification (*p* < 0.05, **Figure 3D** **),** and histological grade (*p* < 0.05, **Figure 3F**). To further explore the relationship between *HMMR* expression and clinicopathological parameters, the clinical data of 316 LUAD patients were obtained from the TCGA database. As depicted in **Table 3**, the high *HMMR* expression level was significantly correlated with gender (*p* = 0.043), pathological stage (*p* = 0.003), T stage (*p* = 0.033), lymph node metastasis (*p* = 0.001), and distant metastasis (*p* = 0.042). In **Table 4**, by adopting logistic regression analysis, we observed that the upregulated expression of *HMMR* mRNA in LUAD was significantly related to gender (OR = 1.594 for male *vs.* female, *p* = 0.012), pathological stage (OR = 2.139 for stage II vs. stage I and *p* = 0.008, OR = 2.316 for stage III *vs.* stage I and *p* = 0.005, OR = 2.574 for stage IV *vs.* stage I and *p* = 0.014), T classification (OR = 1.845 for T2 *vs.* T1 and *p* < 0.001), and lymph node metastasis (OR = 2.195 for positive *vs.* negative and *p* < 0.001).

**Table 3:** Relationships between *HMMR* expression and clinicopathological parameters in LUAD.

Clinicopathological parameters	*HMMR* expression		Total	*p*-value
	High (n=158)	Low (n=158)		
**Age**				
** <65 years**	69 (49.6)	70 (50.4)	139	0.910
** ≥65 years**	89 (50.3)	88 (49.7)	177	
**Gender**				
** Male**	86 (55.8)	68 (44.2)	154	**0.043**
** Female**	72 (44.4)	90 (55.6)	162	
**Pathological stage**				
** I–II**	108 (45.2)	131 (54.8)	239	**0.003**
** III–IV**	50 (64.9)	27 (35.1)	77	
**T classification**				
** T1–T2**	130 (47.6)	143 (52.4)	273	**0.033**
** T3–T4**	28 (65.1)	15 (34.9)	43	
**Lymph node metastasis**				
** Negative**	86 (42.8)	115 (57.2)	201	**0.001**
** Positive**	72 (62.6)	43 (37.4)	115	
**Distant metastasis**				
** No**	143 (48.5)	152 (51.5)	295	**0.042**
** Yes**	15 (71.4)	6 (28.6)	21	

Bold values indicate p < 0.05.

**Table 4:** *HMMR* expression correlated with clinicopathological parameters.

Clinicopathological parameters	Total (*N*)	Odds ratio in *HMMR* expression	*p*-value
**Age**			
** <65 *vs.* ≥65**	458	0.900 (0.624–1.299)	0.575
**Gender**			
** Male *vs.* female**	477	1.594 (1.110–2.295)	**0.012**
**Pathological stage**			
** Stage II *vs.* stage I**	366	2.139 (1.359–3.390)	**0.008**
** Stage III *vs.* stage I**	335	2.316 (1.385–3.926)	**0.005**
** Stage IV *vs.* stage Ⅰ**	282	2.574 (1.117–6.288)	**0.014**
**T classification**			
** T2 *vs.* T1**	414	1.845 (1.236–2.769)	**<0.001**
** T3 *vs.* T1**	200	2.445 (1.220–5.022)	>0.05
** T4 *vs.* T1**	178	2.682 (1.022–7.555)	>0.05
**Lymph node metastasis**			
** Positive *vs.* negative**	465	2.195 (1.485–3.266)	**<0.001**
**Distant metastasis**			
** Yes *vs.* no**	348	2.101 (0.898–5.309)	0.097

Bold values indicate p < 0.05. The significance value has been adjusted by the “Bonferroni correction method” for multiple tests in pathological stage and T classification.

### High *HMMR* Expression Is Related to Poor Survival in Patients With LUAD

**Figure 4:**
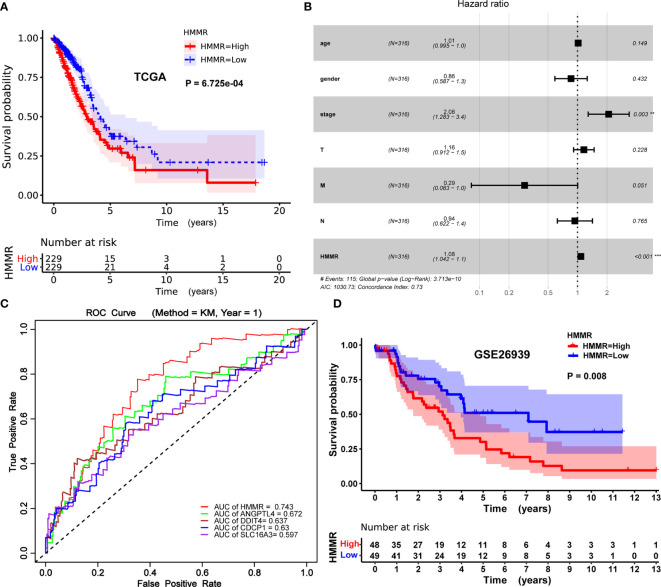
Prognostic role of *HMMR* in LUAD patients. **(A)** Kaplan–Meier curve of the association between *HMMR* mRNA expression and the prognosis of LUAD patients. **(B)** Forest plot of the multivariate Cox proportional hazard regression model indicated that *HMMR* was an independent predictor of poor survival rate (HR: 1.080, 95% CI: 1.042–1.120, *p* = 0.000). **(C)** ROC curves of evaluating the accuracy of each prognostic gene. **(D)** Kaplan–Meier curve of the association between *HMMR* mRNA expression and the prognosis of LUAD patients in GSE26939. Hazard ratio (HR), confidence interval (CI), receiver operating characteristic (ROC), area under the curve (AUC). (*p < 0.05, **p < 0.01, ***p < 0.001).

The Kaplan–Meier risk estimate was applied to evaluate the prognostic role of *HMMR* in LUAD. Compared with the low *HMMR* expression group, the high *HMMR* expression group was more associated with a poor OS (**Figures 4A, D** and **S3**). Besides, the median OS of the high *HMMR* expression group was 34.77 months and the low *HMMR* expression group had a median OS of 49.93 months in **Figure 4A**. The 5-year survival rate of the high *HMMR* expression group (29.7%) was also lower than that of the low *HMMR* expression group (37.5%) in **Figure 4A**. Further, we implemented univariate and multivariate analyses on 316 LUAD patients collected from the TCGA database to investigate the impact of the *HMMR* expression and clinicopathological factors on survival. Univariate analysis revealed four vital predictors of survival including pathological stage (HR: 1.654, 95% CI: 1.401–1.951, *p* < 0.001), T stage (HR: 1.632, 95% CI: 1.315–2.024, *p* < 0.001), N stage (HR: 1.790, 95% CI: 1.459–2.196, *p* < 0.001), and *HMMR* expression (HR: 1.084, 95% CI: 1.050–1.120, *p* < 0.001). Multivariate analysis turned out that the high *HMMR* expression was a crucial independent predictor of a poor OS in LUAD (HR: 1.080, 95% CI: 1.042–1.120, *p* < 0.001) (**Figure 4B** and **Table 5**).

**Table 5:** Univariate and multivariate analyses of the correlation of *HMMR* expression with LUAD patients.

Parameter	Univariate analysis			Multivariate analysis		
	HR	95% CI	*p*	HR	95% CI	*p*
**Age**	1.002	0.983–1.021	0.843	1.015	0.995–1.035	0.149
**Gender**	1.035	0.717–1.494	0.852	0.859	0.587–1.255	0.432
**Pathological stage**	1.654	1.401–1.951	**<0.001**	2.078	1.283–3.365	**0.003**
**T**	1.632	1.315–2.024	**<0.001**	1.160	0.912–1.476	0.228
**N**	1.790	1.459–2.196	**<0.001**	0.939	0.662–1.417	0.765
**M**	1.757	0.964–3.203	0.066	0.288	0.083–1.004	0.051
***HMMR***	1.084	1.050–1.120	**<0.001**	1.080	1.042–1.120	**<0.001**

HR, hazard ratio; CI, confidence interval. Bold values indicate p < 0.05.

To further evaluate the accuracy of *HMMR* as a prognostic gene, we drew the receiver operating characteristic (ROC) curve of each prognostic gene (*HMM*, *ANGPTL4*, *CDCP1*, *DDIT4*, and *SLC16A3*) and calculated the area under the curve (AUC) value of each prognostic gene. The process of obtaining these genes (*ANGPTL4*, *CDCP1*, *DDIT4*, and *SLC16A3*) with prognostic potential has been elaborated in the supplementary material (**Table S2**, **S3**, and **Figure S1**). As described in **Figure 4C**, the performance of *HMMR* is better (AUC = 0.743).

### Identification of *HMMR*-Related Signaling Pathways and 10 Genes Co-Expressed With *HMMR*

**Figure 5:**
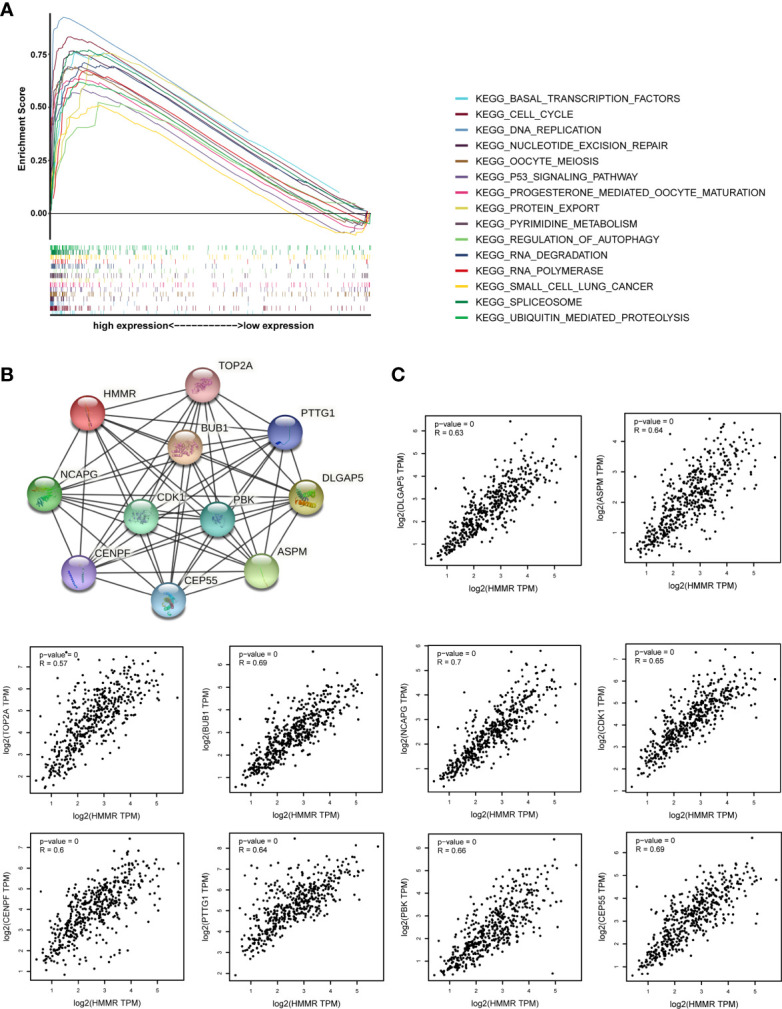
The investigation on the potential mechanism of *HMMR* in promoting LUAD progression. **(A)** The merged enrichment plot including enrichment scores and gene sets. **(B)** The PPI network plot for *HMMR* including 10 predicted genes co-expressed with *HMMR*. **(C)** Correlation between *HMMR* and 10 genes co-expressed with *HMMR* (*TOP2A*, *PTTG1*, *DLGAP5*, *ASPM*, *CEP55*, *CENPF*, *NCAPG*, *BUB1*, *PBK*, and *CDK1*).

GSEA was performed to investigate the possible mechanism of *HMMR* in promoting LUAD progression. We first divided TCGA samples into high and low *HMMR* expression groups. According to the normalized enrichment score (NES), false discovery rate (FDR) *q*-value, and nominal (NOM) *p*-value, 15 significantly enriched signaling pathways with the high *HMMR* expression phenotype were identified and listed as cell cycle, oocyte meiosis, ubiquitin-mediated proteolysis, RNA degradation, basal transcription factors, progesterone-mediated oocyte maturation, pyrimidine metabolism, nucleotide excision repair (NER), spliceosome, p53 signaling pathway, DNA replication, protein export, small cell lung cancer, RNA polymerase, and regulation of autophagy (**Figures 5A**, **S2**, and **Table 6**). Based on the STRING database, we also identified 10 genes co-expressed with *HMMR* (*TOP2A*, *PTTG1*, *DLGAP5*, *ASPM*, *CEP55*, *CENPF*, *NCAPG*, *BUB1*, *PBK*, and *CDK1*) according to the confidence score and built the corresponding PPI network (*p*-value = 8.03e-10) (**Figure 5B**). Moreover, a correlation analysis by GEPIA demonstrated that the mRNA levels of *TOP2A*, *PTTG1*, *DLGAP5*, *ASPM*, *CEP55*, *CENPF*, *NCAPG*, *BUB1*, *PBK*, and *CDK1* were significantly associated with *HMMR*, indicating that *HMMR* may work with these genes to promote LUAD progression (**Figure 5C**).

**Table 6:** Gene sets enriched in the high *HMMR* expression phenotype.

Gene set name	NES	NOM *p*-value	FDR *q*-value
**KEGG_CELL_CYCLE**	2.702	<0.001	<0.001
**KEGG_OOCYTE_MEIOSIS**	2.657	<0.001	<0.001
**KEGG_UBIQUITIN_MEDIATED_PROTEOLYSIS**	2.498	<0.001	<0.001
**KEGG_RNA_DEGRADATION**	2.403	<0.001	<0.001
**KEGG_BASAL_TRANSCRIPTION_FACTORS**	2.388	<0.001	<0.001
**KEGG_PROGESTERONE_MEDIATED** **_OOCYTE_MATURATION**	2.387	<0.001	<0.001
**KEGG_PYRIMIDINE_METABOLISM**	2.382	<0.001	<0.001
**KEGG_NUCLEOTIDE_EXCISION_REPAIR**	2.354	<0.001	<0.001
**KEGG_SPLICEOSOME**	2.342	<0.001	<0.001
**KEGG_P53_SIGNALING_PATHWAY**	2.243	<0.001	<0.001
**KEGG_DNA_REPLICATION**	2.184	<0.001	<0.001
**KEGG_PROTEIN_EXPORT**	2.035	<0.001	0.003
**KEGG_SMALL_CELL_LUNG_CANCER**	1.964	0.004	0.007
**KEGG_RNA_POLYMERASE**	1.946	0.006	0.008
**KEGG_REGULATION_OF_AUTOPHAGY**	1.697	0.008	0.044

NES, normalized enrichment score; NOM, nominal; FDR, false discovery rate.

## Discussion

The research on the role of *HMMR* in occurrence and progression of tumors has recently attracted widespread attention. Lots of studies have declared that *HMMR* is abnormally expressed in different types of cancers. With the growing development of high-throughput sequencing technology, various data of gene expression level in LUAD samples were uploaded to the public database, such as GEO and TCGA, which provides chances for biomarker discovery and validation. In this study, through taking full use of publicly available databases TIMER2.0 and UALCAN, we first analyzed the *HMMR* expression level among various human tumors. These results implied that *HMMR* gene expression was upregulated in lung adenocarcinoma, bladder urothelial carcinoma, breast invasive carcinoma, clear cell renal cell carcinoma, cholangiocarcinoma, esophageal carcinoma, stomach adenocarcinoma, and others than in their matched adjacent normal tissues. Then, we focused on the expression of *HMMR* in lung adenocarcinoma.

In the current study, we have made huge attempts to detect the role of *HMMR* expression in LUAD progression, especially as a prognostic biomarker for LUAD. Additionally, we screened the signal pathways associated with *HMMR* in LUAD to reveal the potential mechanism of *HMMR* regulating LUAD development. First, on the basis of the RNA-seq data from the TCGA cohort, we observed that the *HMMR* expression level in LUAD tissues was significantly higher than that in nontumor tissues and the result has been confirmed in mRNA and protein levels by qRT-PCR and public TMAs. Also, meta-analysis was implemented on the GEO cohort to compare the differences in the *HMMR* expression level between LUAD tissues and nontumor tissues, which obtained the same conclusion (18). These results indicated that *HMMR* may serve as an oncogene and play an important role in LUAD initiation and progression. Moreover, it was observed that the *HMMR* expression level was different in groups classified by pathological stage, T stage, N stage, and M stage. After further analyzing the relationship between *HMMR* expression and clinicopathological parameters, it is shown that the high *HMMR* expression level was significantly correlated with pathological stage, T classification, lymph node metastasis, and distant metastasis. In 2018, Song et al. (20) proposed that the overexpression of *HMMR* in LUAD was correlated with late pathological stage and reduced OS. Later, He et al. (16) put forward that *HMMR* could be regarded as a prognostic signature in early-stage NSCLC. All in all, these studies implied that the *HMMR* expression at the mRNA level is related to some important clinicopathological parameters.

Kaplan–Meier survival analysis indicated that the prognosis of the high *HMMR* expression group was worse than that of the low *HMMR* expression group. Besides, the univariate analysis manifested that the high *HMMR* expression was related to poor OS. Pathological stage, T stage, and N stage were also associated with the prognosis of LUAD patients. In general, we found that *HMMR* could be treated as an independent prognostic factor for the OS of LUAD patients and proved its potential as a biomarker for LUAD.

The GSEA method was utilized to analyze signaling pathways of *HMMR* in LUAD. The results showed that cell cycle, oocyte meiosis, ubiquitin-mediated proteolysis, RNA degradation, basal transcription factors, progesterone-mediated oocyte maturation, pyrimidine metabolism, NER, spliceosome, p53 signaling pathway, DNA replication, protein export, small cell lung cancer, RNA polymerase, and regulation of autophagy were correlated with the progression of LUAD. The operation of the cell cycle is precisely regulated by various factors inside and outside the cell, and internal factors are the basis for regulation. Disorders of the cell cycle and abnormal cell proliferation may lead to cell canceration, and each aspect of the cell cycle regulation system may be the main factor leading to cell canceration (21). The ubiquitin pathway plays a key role in regulating cell growth and proliferation *via* controlling the abundance of cyclins. Besides, unscheduled proteolysis of many cell cycle regulators contributes to tumorigenesis (22). Abnormal degradation of RNA may inhibit gene expression, thereby inducing cancer or promoting cancer progression (23). Basic transcription factors are necessary for the initiation of RNA polymerase II transcription and can maintain the basic level of transcription. Once the basic level of transcription is imbalanced, it will affect the function of RNAs and then induce the occurrence and progression of tumors (24). Moreover, abnormal pyrimidine metabolism plays a certain role in the process of tumor invasion and metastasis (25). NER can eliminate structurally unrelated DNA lesions through a multiwise “cut and patch” reactions. Further, the global genome NER sub-pathway prevents mutagenesis by detecting twisting damages of the genome. Therefore, defects in the global genome NER may cause cancers (26). As an important regulatory step in the process of gene expression, abnormal splicing regulation is a common feature among various cancers. Specifically, these cancers may be caused by mutations that disrupt the splicing of specific genes or by the general loss of spliceosomal function, thereby affecting many gene targets (27). p53 stabilizes the genome by interacting with different signal transduction pathways in cells, thereby regulating various cellular processes. The mutations in p53 have associations with genomic instability and an increased sensitivity to cancers (28). Furthermore, we constructed the PPI network through utilizing the STRING database and gave 10 genes that are likely to co-express with *HMMR*.

This study also has some limitations. First of all, the clinical data lack some important information, such as tumor size. Specific details that are critical to the prognosis of patients, including surgical treatments and surgical details, are not provided. Based on public available databases including GEO and TCGA, we are unable to clarify the direct functional mechanism of *HMMR* in LUAD.

## Conclusion

In conclusion, through analyzing *HMMR* expression data of LUAD patients, we observed that the *HMMR* expression in LUAD tissues is higher than that in nontumor tissues. It is found that the upregulation of *HMMR* is closely correlated with some clinicopathological features of LUAD. We infer that the upregulation of *HMMR* promotes the occurrence and progression of LUAD. According to univariate and multivariate survival analyses, the increased *HMMR* expression in LUAD was identified as an independent risk factor for shorter OS. All in all, we believe that the *HMMR* expression level can become a promising marker for the diagnosis and prognosis of LUAD.

## Data Availability Statement

Available datasets in this study were analyzed and can be downloaded from The Cancer Genome Atlas (https://portal.gdc.cancer.gov/) and the NCBI Gene Expression Omnibus (GSE101929, GSE11969, GSE18842, GSE21933, GSE27262, GSE32863 and GSE75037).

## Ethics Statement

The project was granted approval by the Ethics Committee of the Affiliated Hospital of Xuzhou Medical University. The patients/participants provided their written informed consent to participate in this study.

## Author Contributions

XL and LiZ designed the overall idea of this study, conceived the experiments, analyzed the data, prepared the figures and tables, and authored the drafts of the paper. QS and YX collected the data from the TCGA and GEO datasets and performed the experiments. LoZ and HZ guided and supervised this study and reviewed the drafts of the paper. All authors contributed to the article and approved the submitted version.

## Funding

This study was supported by the Talented Scientific Research Foundation of Xuzhou Medical University (No. D2018018).

## Conflict of Interest

The authors declare that the research was conducted in the absence of any commercial or financial relationships that could be construed as a potential conflict of interest.

## Publisher’s Note

All claims expressed in this article are solely those of the authors and do not necessarily represent those of their affiliated organizations, or those of the publisher, the editors and the reviewers. Any product that may be evaluated in this article, or claim that may be made by its manufacturer, is not guaranteed or endorsed by the publisher.
