# Supplementary material for: Validating HMMR Expression and Its Prognostic Significance in Lung Adenocarcinoma Based on Data Mining and Bioinformatics Methods
Source: Front Oncol. 2021 Aug 30;11:720302. doi: 10.3389/fonc.2021.720302 (PMC8435795; doi:10.3389/fonc.2021.720302)
Supplement: Supplementary file 1 [file DataSheet_1.docx]

Supplementary Material

**Supplementary Table 1.** The difference multiple of *HMMR* expression between normal samples and tumor samples for common cancers.

| Cancer type  (TCGA project) | HMMR  average expression  (normal samples) | | HMMR  average expression  (tumor samples) | Log2FC | p-value |
| --- | --- | --- | --- | --- | --- |
| BLCA | 1.17 (19) | 5.63 (414) | | 2.27 | 1.71e-09 |
| BRCA | 0.53 (113) | 4.17 (1109) | | 2.97 | 1.18e-58 |
| CESC | 0.15 (3) | 7.32 (306) | | 5.63 | 2.91e-03 |
| CHOL | - | - | | - | - |
| COAD | 7.56 (41) | 7.20 (473) | | -0.07 | 0.39 |
| ESCA | 1.76 (11) | 7.84 (160) | | 2.15 | 2.94e-06 |
| GBM | - | - | | - | - |
| HNSC | - | - | | - | - |
| KICH | 0.31 (24) | 0.89 (65) | | 1.50 | 0.07 |
| KIRC | 0.42 (72) | 1.06 (539) | | 1.35 | 1.69e-24 |
| KIRP | 0.20 (32) | 0.79 (289) | | 1.97 | 5.94e-10 |
| LIHC | 0.16 (50) | 2.49 (374) | | **3.99** | 4.54e-27 |
| PAAD | 1.23 (4) | 2.09 (178) | | 0.77 | 0.12 |
| PRAD | 0.41 (52) | 1.10 (499) | | 1.43 | 1.99e-15 |
| PCPG | - | - | | - | - |
| READ | 2.00 (2) | 8.13 (89) | | 2.02 | 0.03 |
| SARC | - | - | | - | - |
| SKCM | 5.99 (1) | 3.55 (471) | | -0.76 | 0.23 |
| THCA | - | - | | - | - |
| THYM | 8.99 (2) | 7.65 (90) | | -0.23 | 0.69 |
| STAD | 1.46 (32) | 6.66 (375) | | 2.18 | 1.74e-15 |
| UCEC | - | - | | - | - |
| LUSC | 0.54 (49) | 5.42 (502) | | **3.32** | 9.37e-30 |
| LUAD | 0.46 (54) | 4.28 (497) | | **3.22** | 4.45e-30 |

Fold change (FC), bold values indicate log2FC>3.

As listed in the Introduction section of our article, a total of 17 genes (*AGRN*, *AKR1A1*, *ANGPTL4*, *B4GALT1*, *CDCP1*, *CIRBP*, *DDIT4*, *EXT1*, *GPC1*, *HLF*, *KBTBD7*, *RBCK1*, *SOD1*, *SEC24B-AS1*, *SH2B1*, *SLC16A3*, *TPX2*) have been reported. We found that there were no expression and clinical data of *EXT1* and *KBTBD7* in our TCGA cohort. Meanwhile, it has been well reported that *TPX2* could promote metastasis and serve as a marker of poor prognosis in non-small cell lung cancer in (1). Therefore, we only compared the biomarker potential of *HMMR* with the remaining 14 genes (*AGRN*, *AKR1A1*, *ANGPTL4*, *B4GALT1*, *CDCP1*, *CIRBP*, *DDIT4*, *GPC1*, *HLF*, *RBCK1*, *SOD1*, *SEC24B-AS1*, *SH2B1*, *SLC16A3*).

Based on TCGA cohort, we first analyzed the expression level of each gene (*AGRN*, *AKR1A1*, *ANGPTL4*, *B4GALT1*, *CDCP1*, *CIRBP*, *DDIT4*, *GPC1*, *HLF*, *RBCK1*, *SOD1*, *SEC24B-AS1*, *SH2B1*, *SLC16A3* and *HMMR*) between normal tissues and LUAD tissues. As shown in **Supplementary Table 2**, the LUAD tissues displayed significantly higher ***HMMR* (log2FC=3.22, *p*=4.45e-30), *ANGPTL4* (log2FC=1.54, *p*=4.49e-07), *CDCP1* (log2FC=1.16, *p*=3.53e-10), *DDIT4* (log2FC=1.20, *p*=1.17e-07) and *SLC16A3* (log2FC=1.51, *p*=2.87e-16)** expression level than the normal tissues.

**Supplementary Table 2.** The expression level of each gene (*AGRN*, *AKR1A1*, *ANGPTL4*, *B4GALT1*, *CDCP1*, *CIRBP*, *DDIT4*, *GPC1*, *HLF*, *RBCK1*, *SOD1*, *SEC24B-AS1*, *SH2B1*, *SLC16A3* and *HMMR*) between normal tissues and LUAD tissues.

| Gene | Average expression (54 normal tissues) | Average expression  (497 tumor tissues) | Log2FC | *p*-value |
| --- | --- | --- | --- | --- |
| AGRN | 27.50 | 44.49 | 0.69 | 1.85e-06 |
| AKR1A1 | 31.12 | 44.45 | 0.51 | 3.08e-11 |
| ANGPTL4 | 8.43 | 24.51 | 1.54 | 4.49e-07 |
| B4GALT1 | 1.37 | 0.76 | -0.85 | 4.01e-19 |
| CDCP1 | 5.86 | 13.05 | 1.16 | 3.53e-10 |
| CIRBP | 43.54 | 31.92 | -0.45 | 1.61e-10 |
| DDIT4 | 29.71 | 68.49 | 1.20 | 1.17e-07 |
| GPC1 | 6.97 | 10.33 | 0.57 | 0.02 |
| HLF | 7.93 | 3.52 | -1.17 | 8.88e-19 |
| RBCK1 | 21.52 | 26.09 | 0.28 | 0.002 |
| SOD1 | 54.84 | 83.80 | 0.61 | 3.84e-12 |
| SEC24B-AS1 | 0.27 | 0.41 | 0.63 | 5.32e-06 |
| SH2B1 | 5.93 | 7.41 | 0.32 | 0.03 |
| SLC16A3 | 8.12 | 23.08 | 1.51 | 2.87e-16 |
| HMMR | **0.46** | **4.28** | **3.22** | **4.45e-30** |

Fold change (FC), bold values indicate log2FC>1.

In **Supplementary Table 2**, it has been observed that *HMMR*, *ANGPTL4*, *CDCP1*, *DDIT4* and *SLC16A3* are overexpressed in LUAD. To further compare the correlation of each gene (*HMMR*, *ANGPTL4*, *CDCP1*, *DDIT4* and *SLC16A3*) expression with LUAD patients, we performed the multivariate analysis. As described in **Supplementary Table 3**, the performance of *HMMR* is better (HR: 1.080, 95% CI: 1.042-1.120, *p*<0.001)

**Supplementary Table 3.** The multivariate analysis of the correlation of each gene (*HMMR*, *ANGPTL4*, *CDCP1*, *DDIT4* and *SLC16A3*) expression with LUAD patients.

| Gene | HR | 95% CI | *p*-value |
| --- | --- | --- | --- |
| ANGPTL4 | 1.005 | 1.000-1.009 | **0.030** |
| CDCP1 | 1.021 | 1.005-1.037 | **0.008** |
| DDIT4 | 1.002 | 1.000-1.005 | **0.036** |
| SLC16A3 | 1.017 | 1.007-1.027 | **0.001** |
| HMMR | 1.080 | 1.042-1.120 | **<0.001** |

Hazard ratio (HR), confidence interval (CI), bold values indicate *p*<0.05.

In **Supplementary Table 2**, it has been observed that *HMMR*, *ANGPTL4*, *CDCP1*, *DDIT4* and *SLC16A3* are overexpressed in LUAD. To further compare the association between each gene (*HMM*, *ANGPTL4*, *CDCP1*, *DDIT4* and *SLC16A3*) mRNA expression and the prognosis of LUAD patients. We conducted the survival analysis experiment. As shown in **Supplementary Figure 1**, the performance of *HMMR* and *SLC16A3* are better.

**Supplementary Figure 1.** Kaplan-Meier curve of the association between each gene (*HMM*, *ANGPTL4*, *CDCP1*, *DDIT4* and *SLC16A3*) mRNA expression and the prognosis of LUAD patients.


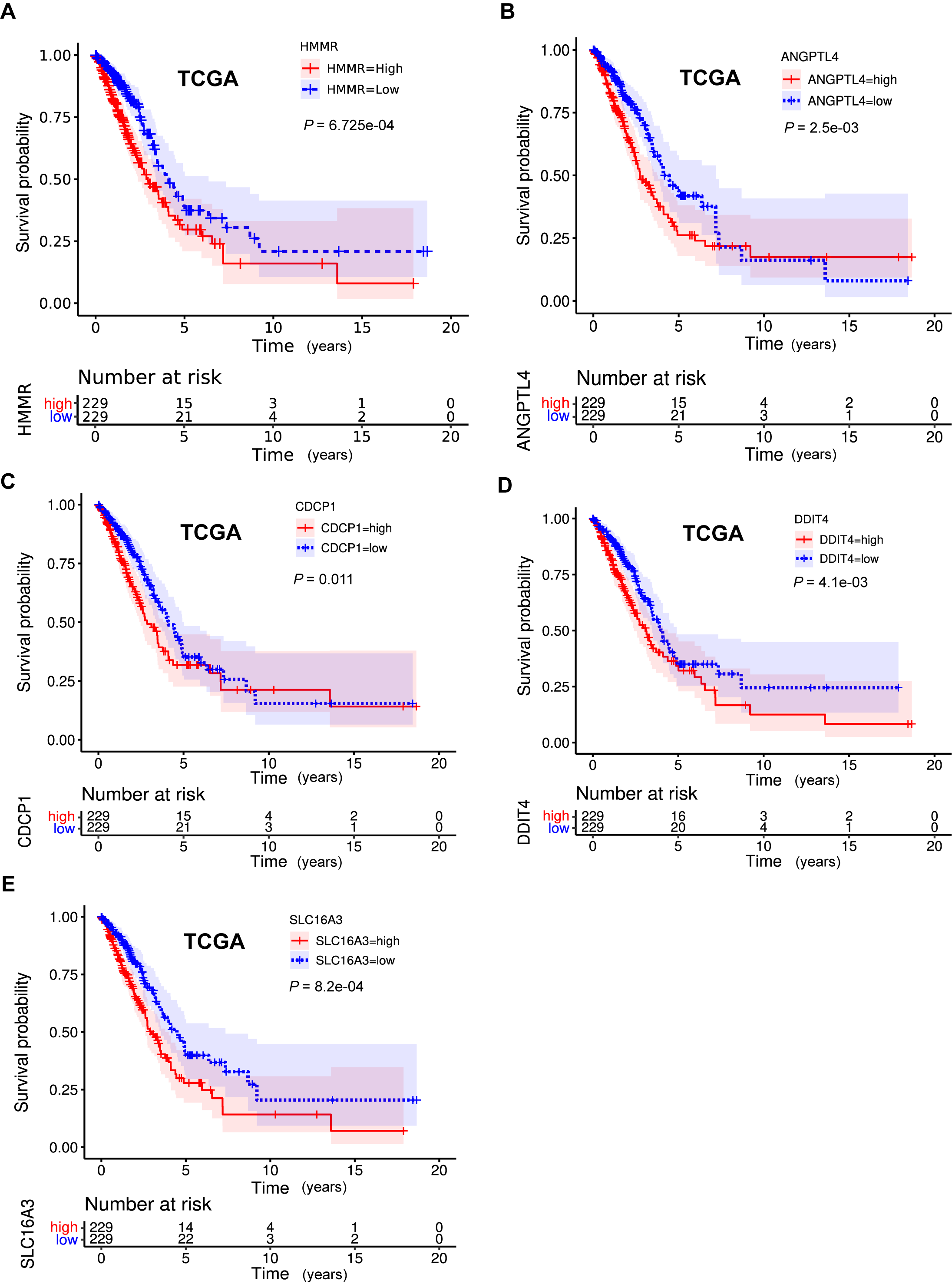


Supplementary Figure 2. Enrichment plots from gene set enrichment analysis. (A) cell cycle, (B) oocyte meiosis, (C) ubiquitin mediated proteolysis, (D) RNA degradation, (E) basal transcription factors, (F) progesterone mediated oocyte maturation, (G) pyrimidine metabolism, (H) nucleotide excision repair, (I) spliceosome, (J) p53 signaling pathway, (K) DNA replication, (L) protein export, (M) small cell lung cancer, (N) RNA polymerase and (O) regulation of autophagy.


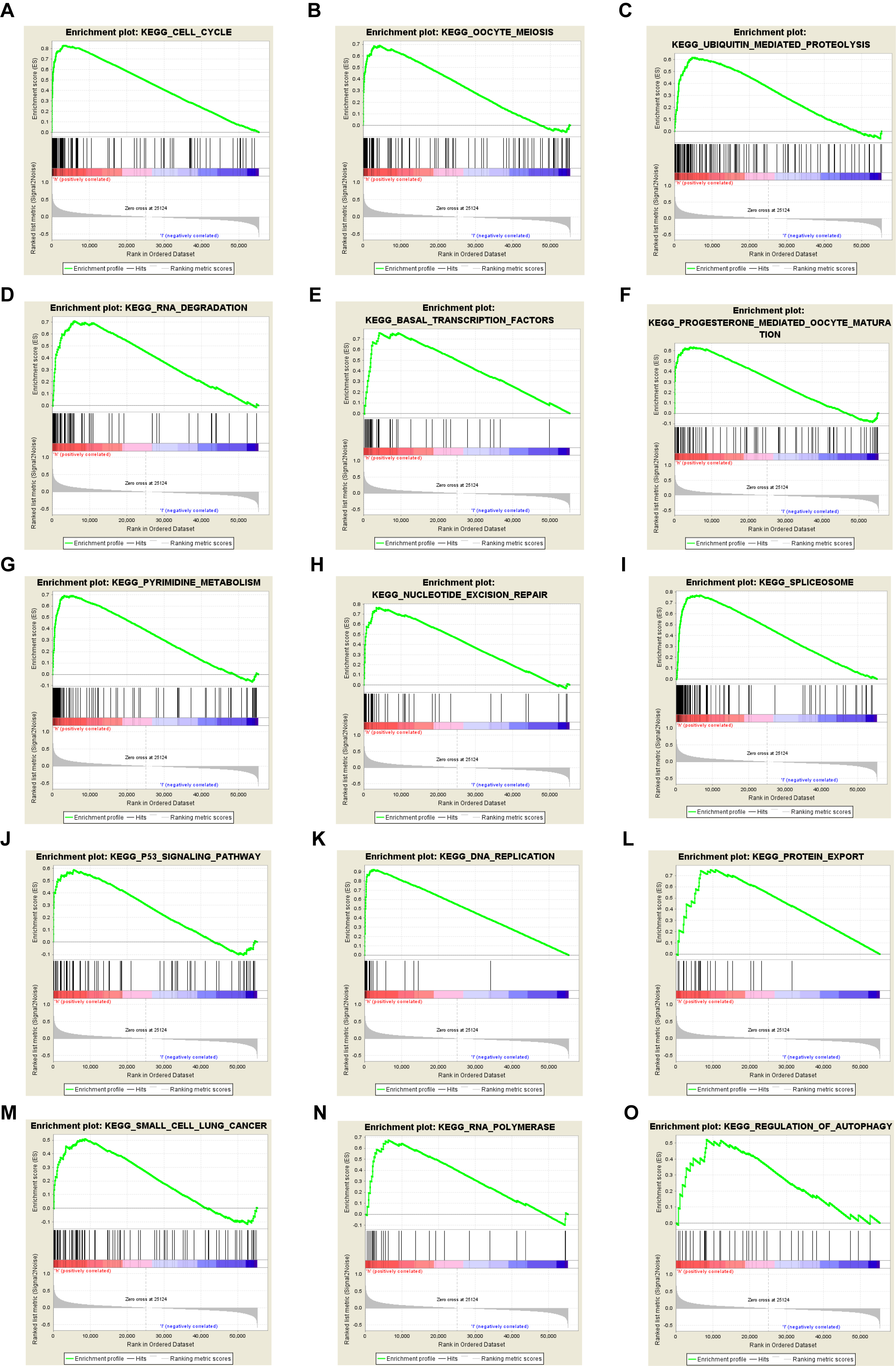


Supplementary Figure 3. Prognostic role of HMMR in LUAD patients based on the k-means clustering model. (A) The sample distribution plot of the high and low HMMR expression group based on the k-means clustering model. (B) Kaplan-Meier curve of the association between HMMR mRNA expression and the prognosis of LUAD patients based on the k-means clustering model.


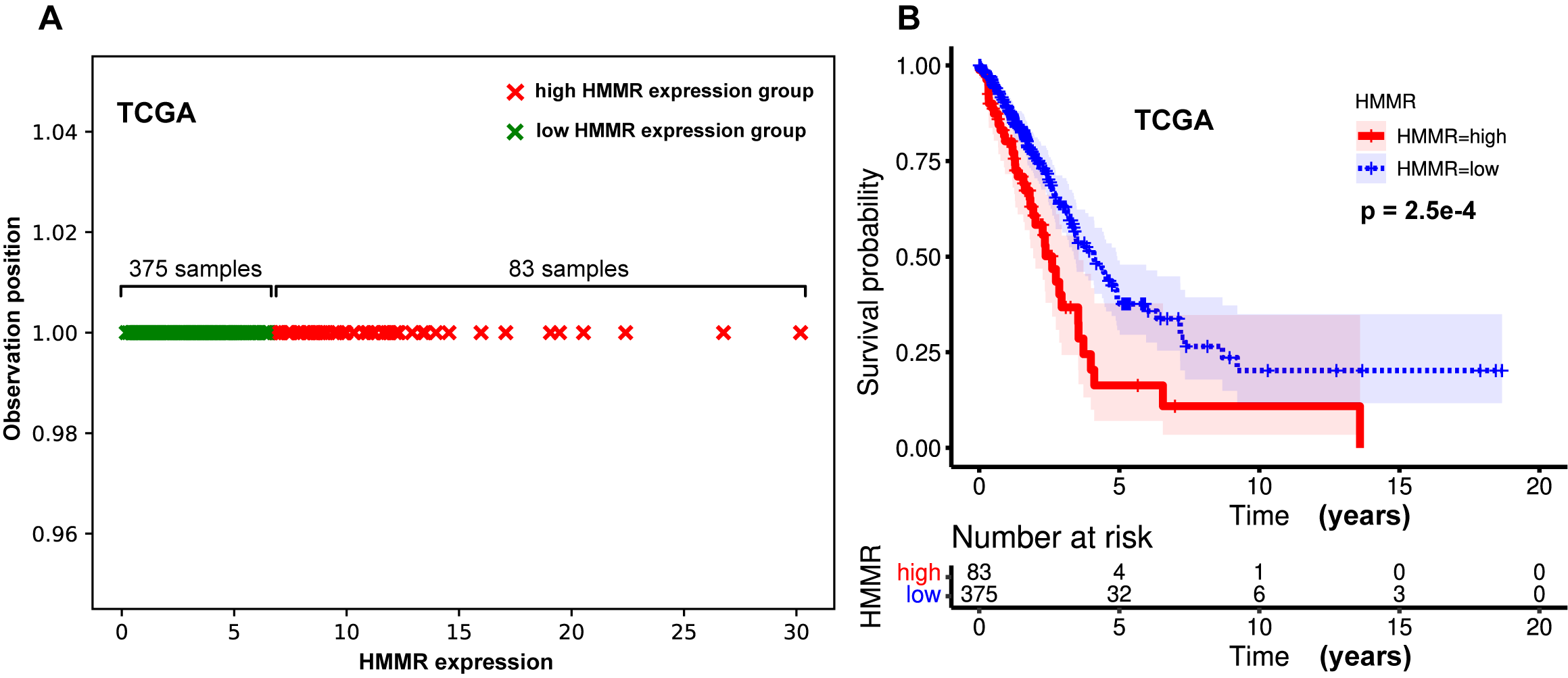


1. Zhou F, Wang M, Aibaidula M, Zhang Z, Aihemaiti A, Aili R, et al. TPX2 Promotes Metastasis and Serves as a Marker of Poor Prognosis in Non-Small Cell Lung Cancer. *Medical science monitor : international medical journal of experimental and clinical research* (2020) 26:e925147. doi: 10.12659/msm.925147.
